# Essays in Preventive Medicine: Glycosuria, and the Period of Infection in Epidemic Diseases

**Published:** 1888-12

**Authors:** 


*Essays in Preventive Medicine: Glycosuria, and the Period of Infection in Epidemic Diseases.* By WILLIAM SQUIRE, M.D. CHURCHILL. 1887.

*Prevention of Infective Fevers, and the Use of Disinfectants, with Notes on the Health of Children.* By WILLIAM SQUIRE, M.D. CHURCHILL. 1887.

*Temperature Observations on Infants and Children in Health and Disease.* By WILLIAM SQUIRE, M.D. CHURCHILL. 1887.

These essays are a record of most interesting and valuable observations by one whose judgment may be trusted. The second of them attempts, for the first time since Gregory's work in 1832, to bring together the facts on "periods of incubation" and "duration of infection." The results are embodied in the following table for each infectious disease:—(1) of the time at which it is likely to show itself after exposure to infection; (2) the time after separation from infection when, if no illness appears, we may conclude the disease has been escaped; (3) the time for the rash or other characteristic sign of the illness to appear after the first sickening for it; (4) the time from the beginning of the illness to the end of infectiveness.

| *Name of Disease.* | *Usual Time of Incubation.* (1) | *Possible Interval.* (2) | *For Rash or other Sign.* (3) | *Duration of Infectiveness.* (4) |
|---|---|---|---|---|
| Small Pox ... | 12 days ... | 15 days ... | 2 to 3 days ... | 3 to 4 weeks |
| Measles ... ... | 8 to 12 ,, ... | 18 ,, ... | 3 to 4 ,, ... | 3 to 4 ,, |
| Scarlet Fever... | 2 to 5 ,, ... | 8 ,, ... | 2 ,, ... | 6 to 8 ,, |
| Rubella ... ... | 10 to 21 ,, ... | 3 weeks... | 1 to 2 ,, ... | 2 to 3 ,, |
| Chicken Pox ... | 10 to 14 ,, ... | 2½ ,, ... | 1 ,, ... | 2 ,, |
| Mumps ... ... | 12 to 21 ,, ... | 3 ,, ... | 1 to 5 ,, ... | 2 or 3 ,, |
| Whooping Cough | 6 to 12 ,, ... | 2 ,, ... | 1 to 3 weeks... | 6 ,, more or less |
| Diphtheria ... | 2 to 12 ,, ... | 2 ,, ... | 1 to 2 days ... | 6 or 8 weeks |
| Enteric Fever | 5 to 20 ,, ... | a month ... or more | 10 to 12 ,, ... | 4 to 8 ,, or more |
| Typhus Fever | 2 to 12 ,, ... | fortnight ... | 5 ,, ... | 3 or 4 weeks |
| Cholera ... ... | 2 to 5 ,, ... | a week ... | sudden ... | uncertain |

The above table illustrates the kind of work which the author records in his essays, which are full of information, and are well worthy of careful study.

